# Death Ascribed to the Use of Chloroform at the Massachusetts General Hospital

**Published:** 1853-01

**Authors:** 


### EDITORIAL DEPARTMENT.

---

*Death ascribed to the use of Chloroform at the Massachusetts General Hospital.*—In the New Hampshire Journal of Medicine, we find a paper copied from the Boston Traveler, read by Dr. J. C. Warren, before the medical class, in which death in one case, and serious symptoms in another case, are attributed to the use of chloroform administered at the Mass. Gen. Hospital in connection with surgical operations.

It may, perhaps, be known to our readers, that Dr. Warren, early in the early history of chloroform, took ground against that anæsthetic agent, and recommended in its stead chloric ether. He has contended for the danger of the former, and the freedom from risk attending the employment of the latter. Under these circumstances, it is singular that chloroform should have been administered, *by mistake*, on the day on which the accidents just referred to occurred. "Chloroform," the Dr. states, "the popular favorite, is never used in this institution (Mass. Gen. Hospital) except as an external application; but on this occasion it was introduced from the fact that it had been poured into a bottle labled with the title, 'concentrated chloric ether.' This bottle, then, marked as concentrated chloric ether, was placed on the table, and employed for these operations without suspicion on our part that it was not the article designated by the label on the surface. The error escaped the observation of those who administered it from the fact that there is a resemblance in the sensible qualities of the two articles."

We do not find in the Med. Journal referred to the evidence that the article was not chloric ether. This, we think, it would have been well to have introduced into the paper.

We give the account of the operations, etc., in full, and in the language of the operator:

I shall now proceed to give an account of three cases in which chloroform was accidentally administered in this hospital on last Saturday. In my account, I shall endeavor to avoid the use of names, as more delicate and proper; but I would say, that all who aided me on this occasion performed their duties with activity and with credit.

Three cases presented themselves for operation. The first was on a contracted hand. The patient was etherized with the supposed chloric ether. In two or three minutes, anæsthesia being produced, the operation was performed under the continued administration of the same article during from five to ten minutes. The patient escaped without any other inconvenience than a slight soreness of the throat—an effect of the inhalation of chloroform which I have myself experienced.

The second case was of a tumor on the right side of the face in the region of the parotid gland, supposed to lie in the substance of this gland. The attendants being arranged so as to give every assistance to the patient and the operator, the anæsthetical liquid was applied as usual with a sponge. and with the freedom employed in the use of ether, but not proper where chloroform is known to be used. As soon as the application was made, the patient began to struggle and throw his limbs about in so violent a manner that we were compelled for the moment to resign him into the hands of the assistants. But being soon exhausted by the excessive motion, he necessarily inhaled more freely, thus filling his lungs with the vapor of chloroform, and in three or four minutes rendering him insensible. The operation was begun, the parotid gland laid bare, the tumor found to lie behind it, the parotid gland itself incised, and a round regular tumor, enclosed in a fibrous sac, was brought into view. At this moment those appointed to watch the patient gave a signal that the pulse was failing and respiration scarcely perceptible. Immediately cold water was dashed on his face, and this not reviving him, motions of the chest (in imitation of respiration,) by moving the ribs up and down, blowing of air into his face, and afterwards into one nostril, stopping the other, (pressing back the larynx, so as to prevent the air from going into the stomach,) frictions of the limbs, clearing the mouth of saliva and froth, and removing the mucus from the throat by the finger were resorted to. At this period, I called for ammonia. During the past year I have been in the habit when a patient was to be etherized, to direct a bottle of ammoniated alcohol to be placed on the table. This substance is a powerful stimulant, and has been employed from an ancient period to revive persons affected with many of the forms of suffocation, or asphyxia, and also for fainting. Its object is not to oxygenate the blood, which it cannot do, but to give a spur to the nervous system, and put in motion the dormant vital energy, for which purpose it is superior to any other stimulant. This is not the strongest preparation, but there was brought by mistake, and without my being aware of the fact, a bottle of aqua ammoniæ, which is three times stronger than the above named. It has the same color, and also the same odor, though the latter in a greater degree, with the ammoniated alcohol. The difference, however, would not be detected in a case of urgency, where a few seconds lost might be fatal to the patient. This article then was applied on a sponge to the nostrils, and not producing any effect, a small portion was insinuated into the mouth. At this moment some faint appearance of respiration was exhibited, and by the continued efforts of artificial respiration and frictions the pulse returned, the patient began to breathe freely, and in from five to ten minutes more he seemed quite out of danger. The operation was then concluded, and the patient carried to his bed.

After lying a few hours he recovered his usual state, at least so far as to speak and drink without difficulty; he was, however, much troubled with a secretion of mucus from the lungs, and a cough necessary to extricate it. He had also soreness of the throat in swallowing. The latter symptom has at this time disappeared, but the cough and expectoration continue. The patient wishes to leave the hospital this afternoon, but I shall previously take occcasion to call him into your presence.

The third case is the most important. It was that of a young man, about twenty years old, a native of Ireland, who had his arm entangled in the machinery of a bark mill about five days before. The muscles and other organs were torn from the fore part of the arm, and some loss of blood took place. On his entrance to the hospital the hand was found cold and without sensation, showing that the nerves had been destroyed, and that his arm could not be restored. Amputation was proposed to him, but he rejected it, and notwithstanding the danger of mortification and lockjaw, and the ultimate uselessness of the limb were pointed out to him, he insisted that he would die with his arm on.

On Saturday, partial mortification having taken place in the meantime, rendering the arm excessively painful and fœtid, and being convinced that he could never recover the use of it, even if he lived, he agreed to have it amputated. The surgeons and assistants took their places around him, as in the last case, while I myself watched his pulse. Etherization was carefully made; immediately on the application of the anæsthetic fluid he became perfectly quiet; the operation proceeded and was accomplished in about two minutes. Just as it was finished, I perceived his pulse was rapidly failing. Word was given to suspend the dressing, and dash water on his face, which was immediately done. Notwithstanding this, the respiration and pulse went on diminishing, and soon ceased. He was, to all appearance, entirely dead. Artificial respiration was directly produced by moving the ribs; the limbs were rubbed, ammonia was momentarily applied to the nostrils and mouth, and when these things failed ammonia was introduced into the mouth as in the other case. Soon after this, to our great joy, a slight inspiration followed, and, the efforts being continued, his respiration improved, though he breathed with difficulty, owing to the quantity of mucus in the lungs. By great efforts on the part of the gentlemen standing around in lifting and turning him on his side, so as to drain out the mucus from his lungs, and by frequently sponging the back part of his mouth, he was from time to time relieved. At last, passing an empty spoon into his mouth, and pouring some brandy and water from another into it, he was made to swallow fully. A stimulating injection into the bowels was also administered.

After aiding in clearing his lungs for some length of time, it was thought he might be removed to his bed; there a little brandy and water was given occasionally, which he swallowed readily. He also spoke and answered all questions proposed to him until the last moment, showing that the organ of voice was not injured. When asked if he suffered, he said yes, and placed his hand on in the region of the heart. Mucus continued to fill his throat. There was no obstruction in the opening of the larynx, for mucus issued from it in a copious stream, showing that his whole lungs were affected. Having remained with him until the pulse had become pretty good, and the respiration apparently better, we adjourned to meet again in an hour and a half. Placing at the same time the house surgeon at his side with instruction to keep his throat clear of mucus, and support him by stimulants, with the strongest injunctions not to leave him till our return.

Shortly before the time fixed for the return of the surgeons, which was half-past three o'clock, the house surgeon perceiving his pulse to suddenly fail, and that his breathing was more hurried, uncovered the stump to see if it was bleeding, and found some effusion of venous blood, probably produced by the liquefaction of blood from the chloroform poison. He then cleared the mouth of mucus, which he hardly completed when the patient breathed his last without any effort or convulsion. Soon after, an opening was made in the trachea and air blown into the lungs for the purpose of inflation, but without effect. A proposal had been made to do this during life, but it was objected to, because air had already been thrown into the lungs through the nostril, because there was no obstruction in the larynx, because blood might escape through the aperture into the trachea and combine with congestion and mucus in the lungs to increase the difficulty.

On the following morning an examination of the body was proposed, but his friends arriving objected, and although we urged the importance of ascertaining the immediate cause of death, they continued to object decidedly.

*Remarks.* Immediately after the occurrence of alarming symptoms in this case, it was discovered that the substance which had been used was not chloric ether, but chloroform; and not till then did we understand the extraordinary phenomena which presented themselves in this and the preceding case. This patient died with the usual phenomena of chloroform poison.

If we consult the records of fatal cases of chloroform, published by me in 1849, we shall perceive that of fifteen cases there mentioned, the principal part took place in a very sudden manner, some of them occurring a minute or two after the application, and some of them in a period from ten minutes to fifty hours. In the latter cases the lungs were remarkably congested or filled with blood, owing to the poison applied to the air-cells of the lungs, or circulating with the sanguineous fluid, as in asphyxia. From various causes, asphyxia is of frequent occurrence; the phenomena are the same with those presented in these cases, and the remedies are the same — hence the great importance of being well acquainted with the treatment adopted in all such casualties.

The first class seem to have perished almost as if they had been struck by lightning, the powers of the nervous system appearing to be at once annihilated. In the second class the lungs exhibited a most remarkable state of congestion. In the death at ten minutes after the application, the lungs were "a good deal congested, and discharged, when cut, a large quantity of bloody serum." In the death in three quarters of an hour, no examination was made, but "the respiration was infrequent and sighing," showing that the function of the lungs was interrupted. In the third case, "the lungs were filled with blood and softened; bloody serum in pleura." In some of the cases the heart was found disordered, in others the brain, but in the whole number, I believe, without exception, the lungs were charged with blood, or congested—the common, decided effect of chloroform.

The revival from anæsthetic symptoms and prolongation of life for ten minutes, three quarters of an hour and fifty hours, bring these cases into the same category with ours. The vital principle, after appearing to be extinguished, lights up, and gives the hope of recovery, but the blood continuing to accumulate in the lungs from the effect of the poison, and the weakness of the patient, its oxygenation is prevented, and, from want of the animating principle, life is suffocated and extinguished.

Is the fatal termination of the third case to be attributed to any cause other than that which exists in the preceding case? There is no reason to believe this to be the fact. The sinking after revival might lead to a suspicion that some other than the usual cause, congestion of the lungs, existed; and some one has suggested that it might have arisen from ammonia having entered the lungs. How could it be the cause of death in this case? By being introduced into the lungs and irritating and burning these organs? This was impossible in the given circumstances; the patient neither swallowed nor breathed after the ammonia had been employed until the whole had been washed out of the mouth by the abundant mucus. It may have been thought that the ammonia irritated the opening into the larynx and swelled it so that no air could pass through. The air did pass through freely till he died, and so did the mucus, a less volatile fluid than air. Further, when ammonia is introduced into the mouth, its entrance into the larynx is repelled by contraction of the laryngeal muscles, so that it cannot enter. Moreover, it was in excessively small quantity. A saturated solution of nitrate of silver, it may be remarked, is frequently introduced into the mouth and even larynx for curative purposes. Most of the ammonia immediately ran out, and the rest was thrown off by the mucus of the lungs and throat. Had any disorganizing effect been produced, how could the patient have swallowed repeated draughts of brandy up to the period of his death? Finally, if the ammonia destroyed the last patient, how did the second escape the action of the same cause? He used the same quantity, so far as can be judged, he was able to swallow through the day and ever since, and to take even solid food; true, he was stronger than the other, but this difference of strength would have made no difference in the chemical or even vital action, more favorable to the one than to the other; yet the one is well, the other is dead. But I will insist no further on this point, and perhaps have already said more than was required.

We believe that the temporary resuscitation of one of these individuals, and permanent restoration of the other from apparent death must be considered as a triumph honorable to medical science and to this institution.

Several points relating to the foregoing report will be likely to arrest the reader's attention:

1. The occurrence of the mistake of labelling chloroform, "concentrated chloric ether."

2. The occurrence of serious accidents in *two* of *three* cases in which chloroform was administered, and a fatal result in *one* of the cases. That there is a liability to the occurrence of grave symptoms and death from the administration of chloroform is not to be doubted, but that *two* out of *three* patients should be seriously affected, and *one* die, would certainly not have been anticipated from the result of the employment of this anæsthetic agent, in a very extended series of cases in the hands of a great number of surgeons. The reader may fairly doubt if, assuming that the unfortunate events in these cases were due to the chloroform. The administration was conducted with that regard to safety which is proper to be observed with respect to the use of any anæsthetic agent.

3. The occurrence of the *two* mistakes is extraordinary. It seems that *aqua ammoniæ* was employed to revive the patients in lieu of *ammoniated alcohol*, and that the former substance was introduced into the mouth! In connection with this second mistake we are much mistaken if the reader is fully satisfied that the unpleasant after-symptoms in one case, and the death in the other case were not attributable to the inhalation of the vapor of the ammonia. We are free to say that the arguments against this supposition advanced by the Reporter are far from being satisfactory to our mind. It is in our view entirely inadequate to say that "most of the ammoniæ ran out, and the rest was thrown off by the mucus of the lungs and throat!" Let Dr. W. try the experiment, in his own person, of introducing a little aquaammoniæ into the mouth, and he will find, we reckon, that some time will elapse ere the mucus of the lungs and throat wash out the irritating, asphyxiating vapor! He says that the glattis could not have been obstructed because the air and mucus passed freely through it till the patient died. The poisonous action of the vapor of ammoniæ may have been on the lungs rather than the glottis; still, as no examination of the body was made, and *the mode of dying is not given in the report*, it is difficult to form an opinion as to how far the glottis may have been obstructed by œdema occurring shortly before death. In respect to the account of the symptoms preceding death, there is a remarkable deficiency of precision. That a saturated solution of nitrate of silver applied to the larynx does not cause fatal, or unpleasant results, we take to be no ground for arguing that the vapor of ammonia is equally inocuous! This mode of reasoning, it must be admitted, is peculiar. That one person escaped death by the ammonia, is assuredly no argument against a fatal result being due to the ammonia in the other case. Apply the same rule of logic to the chloroform, and it proves incontrovertibly that this agent did not kill the patient in Dr. Warren's case; for chloroform has been given in more than three thousand successive cases without any unpleasant consequences. This argument proves quite too much for Dr. Warren's purpose.

The only point which in the imperfect history of the symptoms is prominent, is the copious secretion of mucus. To quote the language of the report: "Mucus continued to fill his throat. There was no obstruction in the opening of the larynx, *for mucus issued from it in a constant stream*." (Our italics.) "By great efforts on the part of the gentlemen standing around, in lifting and turning him on his side, *so as to drain out the mucus from the lungs*,"* etc. (Our italics.) Now, in view of the facts with respect to the secretion of mucus which are contained in the above quotations, one of two things is clear: either the patient, prior to the operation, was laboring under a pulmonary affection rendering the administration of any anæsthetic agent improper; or the bronchial mucous membrane was exposed to a cause of irritation far more prompt and potent in its operation, than the vapor of chloroform. We can readily enough conceive that the vapor of ammonia would prove an irritating cause adequate to the production of the phenomena.

4. Our contemporary of the New Hampshire Med. Journ., comments on the fact that this report was communicated for the newspaper press. He

* The reader, we fancy, will be surprised to know that by turning a patient on the side, mucus can be drained away from the lungs!

says: "How does it happen that this account of the case appears in a newspaper, instead of a medical Journal, when the third section of the first article of the second chapter of the national code of ethics, says, "it is derogatory to the dignity of the profession to publish cases and operations in the daily prints, or suffer such publication to be made?" We concur fully in the above criticism, and the course pursued is the more censurable, as it seems to us, in view of peculiar circumstances attending the cases reported. Dr. Warren evidently wishes it to be believed that the death was owing to chloroform exclusively. He is excusable for wishing to arrive at this conclusion, and, if he so persuades himself, to desire that others should concur with him. But the question is an open one. He virtually admits that it is so, when he enters in his report upon the discussion of it. This being the case, Dr. Warren should have addressed his arguments and appeal to the medical profession through some appropriate channel, nor to the public, whom, it is to be presumed, are not generally competent to judge respecting matters so purely medical in their nature. We are entitled to *our* opinion on this point, and we do not hesitate to say that, in our judgment, the course pursued should be looked upon as highly improper.

Again, Dr. Warren has, from the first, repudiated in his surgical practice the use of chloroform, but is identified with the suggestion of chloric ether as the only proper and safe anæsthetic agent. In this position Dr. Warren is somewhat singular. At all events, very many, if not most distinguished surgeons, as well as accoucheurs, and general practitioners, are accustomed to use chloroform, and consider it not only more eligible, but a safe article, if properly managed. Now, what will be likely to be the effect of Dr. Warren's report on the public mind in the part of the country where his reputation gives to his opinions more or less influence? It is plainly calculated to create a distrust of chloroform, a fear of it, and a disposition to attach blame to those who employ it; and this, not as the consequence of a candid examination of facts, but as ths result of Dr. Warren's personal influence. Regarded in this point of view, the publication of Dr. Warren's report in the newspapers, prior to any discussion, or action thereon by the medical profession, seems to us unworthy a high-minded practitioner.

As respects the subject of chloroform, we have personally no more interest than most practitioners of medicine. In its relations to medical science and to humanity, all have an interest in this subject; and the report which we have briefly criticised appears to us to be an attempt to create a popular prejudice against the use of chloroform unwarranted by the facts as presented by the reporter himself, and involving a procedure illegitimate and censurable.

